# The effectiveness of a nonalcoholic disinfectant containing metal ions, with broad antimicrobial activity

**DOI:** 10.1038/s41598-020-80443-6

**Published:** 2021-01-13

**Authors:** Tokuhiro Matsubara, Shuichi Maki, Yukiko Toshimori

**Affiliations:** 1 Department of Gastroenterology, Tsukaguchi Hospital, 6-8-1 Minamitsukaguchi-cho, Amagasaki, Hyogo 661-0012 Japan; 2 Yui-Nozomi Hospital, 95 Fushimido, Tondabayashi, Osaka 584-0055 Japan; 3 Shiniryouzaidan, 3-7-4 Matsuzaki-cho, Abeno-ku, Osaka, 545-0053 Japan

**Keywords:** Antimicrobials, Antiviral agents

## Abstract

Disinfectants have different efficacies depending on their use and the target microorganism. This study aimed to evaluate the efficacy and safety of our new nonalcoholic disinfectant, which consists mainly of metal ions. According to the 17th revised Japanese Pharmacopoeia and ASTM international E1052 method, the bactericidal and virucidal efficacy of this new disinfectant against 13 microorganisms was evaluated by the in vitro quantitative suspension test. Additionally, the disinfectant cytotoxicity against multiple cell lines was examined. Then, a safety test using a human open patch test was performed with 26 healthy volunteers. This disinfectant showed strong bactericidal and virucidal activities: all microorganisms except enterovirus were inactivated very quickly. The infectivity of 12 microbial strains was eliminated within 5 min of disinfectant exposure. Additionally, this disinfectant showed little acute cytotoxicity in vitro. All volunteers were negative in the human open patch test. Our new disinfectant has a broad spectrum of microbial targets, is safe for human skin, and demonstrates no cytotoxicity. This disinfectant could prevent common microbial infections.

## Introduction

The spread of nosocomial pathogens, which is a major source of healthcare-associated infections (HAIs), contributes to patient morbidity and mortality^1^. Rutala and Weber^2^ listed the most prevalent pathogens causing HAIs. An estimated 20–40% of HAIs have been supposed to cross-infect from direct contact with patients or indirect contact with contaminated environmental surfaces via the hands of healthcare personnel^3^. Additionally, several viruses with pandemic capacity have emerged in recent years. Pandemics of severe acute respiratory syndrome coronavirus (SARS-CoV) in 2003, H1N1 influenza virus in 2009, middle east respiratory syndrome coronavirus in 2015, SARS-CoV-2 (COVID-19) in 2019, and avian H7N9 influenza virus transmitted from avian to human in 2013 have caused serious economic and social disturbances worldwide^4–9^. Generally, contaminated surfaces are an established common transmission route for several viruses causing pandemics and life-threatening nosocomial pathogens, including *Staphylococcus aureus (S. aureus)*, *Escherichia coli (E. coli)*, *Clostridium difficile* (*C. difficile*) spores, methicillin-resistant *Staphylococcus aureus* (MRSA), vancomycin-resistant enterococci (VRE), norovirus and *Acinetobacter baumannii*, which can survive on surfaces for extended periods^10,11^.

Donskey^12^ reviewed the scientific literature and reported that improving surface cleaning and disinfection reduces the incidence of HAIs. Furthermore, guidelines by the Centers for Disease Control and Prevention, the Food and Drug Administration, the Environmental Protection Agency (EPA), and the International Scientific Forum on Home Hygiene acknowledge the incidence of disease due to insufficient disinfection and that one of the means for the prevention of disease is proper disinfection^10^. However, the selection of proper disinfectants according to the situation is often difficult. Rutala and Weber^2^ summarized the properties of an ideal disinfectant. It is desirable to use a high level of a disinfectant that has these properties, and it is recommended to select a disinfectant with a longer contact time to kill a broader spectrum of microorganisms. To date, a product that meets all the characteristics has not been introduced.

A former disinfectant of ours, reported by Takakuwa et al*.*^13^, consists mainly of one type of iron ion and was shown to have very strong anti-influenza viral activity (including avian, swine, and human), and the acute cytotoxicity was much weaker than that of chlorhexidine gluconate. However, this product has a faint metallic odor and has not yet been evaluated for safety to humans and antimicrobial activity against bacteria and other viruses. Accordingly, we made further improvements to create a new disinfectant with an unprecedented composition containing three types of metal ions. Therefore, this study aimed to evaluate its efficacy against microorganisms and safety for the human skin.

## Results

The results of experiment 1 are presented in Table 1. The number of bacteria after 5 and 30 min of disinfectant exposure was less than the lower limit of quantification or < 100 CFU/mL for the 9 bacteria. Additionally, the log10 reduction value (LRV) was 3.3–4.8, indicating bactericidal efficacy, and the disinfectant is fast-acting and persistent and has a broad antibacterial spectrum. Next, the viral infection titers of influenza A virus and human adenovirus after 15 s and 5 min of exposure to the disinfectant were less than the lower limit of quantification (< 1.3 × 10^1^ the 50% tissue culture infectious dose (TCID_50_)/mL), and those of feline calicivirus were 1.9 × 10^2^ TCID_50_/mL, also less than the lower limit of quantification. Additionally, the LRV was 4.6 to 5.9, indicating virucidal efficacy for the above 3 viruses; however, the viral infection titers of human enterovirus after 15 s and 5 min were 1.5 × 10^4^ TCID_50_/mL and 2.7 × 10^3^ TCID_50/_mL, respectively, and the LRV was 0–0.8, indicating no virucidal efficacy for human enterovirus. Then, the ratios of the bacterial number after the action of the inactivators to that of the control were 92–127%, which were within the criteria in the JP17 (Supplementary Table S1). As a result, the inactivator/neutralizer was validated as being effective in quenching the activity of the biocide, as bacterial viability remained within one-half to 2 times the control value.

**Table 1:** Time taken to completely inactivate microorganisms following contact with the disinfectant.

Pathogens		Reagents	Reaction time			LRV	
			0 min	5 min	30 min	5 min	30 min
			Number of bacteria (CFU/mL)				
#1	*Escherichia coli* NBRC3972	0.85% NaCl	5.2 × 10^5^	4.5 × 10^5^	4.3 × 10^5^		
		Disinfectant		< 10	< 10	> 4.7	> 4.7
#2	*Escherichia coli* (157: H7)	0.85% NaCl	3.4 × 10^5^	5.0 × 10^5^	2.9 × 10^5^		
		Disinfectant		< 10	< 10	> 4.5	> 4.5
#3	*Pseudomonas aeruginosa* NBRC13275	0.85% NaCl	5.4 × 10^5^	6.6 × 10^5^	5.1 × 10^5^		
		Disinfectant		< 10	< 10	> 4.7	> 4.7
#4	*Salmonella enterica* subsp. *enterica* NBRC3313	0.85% NaCl	6.2 × 10^5^	7.4 × 10^5^	6.2 × 10^5^		
		Disinfectant		< 10	< 10	> 4.7	> 4.7
#5	*Staphylococcus aureus* NBRC12732	0.85% NaCl	5.0 × 10^5^	5.6 × 10^5^	4.8 × 10^5^		
		Disinfectant		< 10	< 10	> 4.6	> 4.6
*#6*	*Staphylococcus aureus* (MRSA) IID1677	0.85% NaCl	5.9 × 10^5^	4.4 × 10^5^	3.4 × 10^5^		
		Disinfectant		< 10	< 10	> 4.7	> 4.7
*#7*	*Vibrio parahaemolyticus* NBRC12711	3% NaCl	4.6 × 10^5^	3.9 × 10^5^			
		Disinfectant		< 10		> 4.6	
*#8*	*Campylobacter jejuni* subsp. *jejuni* JCM2013	0.85% NaCl	2.3 × 10^5^	2.0 × 10^5^			
		Disinfectant		< 100		> 3.3	
*#9*	*Candida albicans* NBRC1594	0.85% NaCl	6.7 × 10^5^	6.2 × 10^5^	7.6 × 10^5^		
		Disinfectant		< 10	< 10	> 4.8	> 4.8

Pathogens		Reagents	Reaction time			LRV	
			0	15 s	5 min	15 s	5 min
			Viral infection titer (TCID_50_/mL)				
#10	Influenza A virus, H1N1, A/PR/8/34, ATCC VR-1469	PBS	8.9 × 10^6^		2.4 × 10^6^		0.5
		Disinfectant		< 1.3 × 10^1^	< 1.3 × 10^1^	> 5.8	> 5.8
#11	Feline calicivirus, F-9, ATCC VR-782	PBS	1.1 × 10^7^		1.5 × 10^7^		-0.1
		Disinfectant		1.9 × 10^2^	< 1.3 × 10^1^	4.7	> 5.9
#12	Human adenovirus 5, Adenoid 75, ATCC VR-5	PBS	5.9 × 10^5^		9.5 × 10^5^		-0.2
		Disinfectant		< 1.3 × 10^1^	< 1.3 × 10^1^	> 4.6	> 4.6
#13	Human enterovirus 71, H, ATCC VR-1432	PBS	1.8 × 10^4^		1.8 × 10^4^		0
		Disinfectant		1.5 × 10^4^	2.7 × 10^3^	0	0.8

*CFU/mL* colony-forming units per milliliter, *lower limit of quantification* 10 CFU/mL (except for *Campylobacter*, with a lower limit of quantification of 100 CFU/mL), *min* minutes, *LRV* log reduction value, *TCID*_*50*_*/mL* 50% tissue culture infectious dose.

The results of experiment 2 are presented in Table 2. The viable rate of cells cultured using the cytotoxicity confirmation sample was 50% or greater, and the disinfectant had no cytotoxicity against the cells used for the infectivity titer determination. For A549 cells, the stock solution of the cytotoxicity confirmation sample had a viable cell rate of less than 50% by absorbance measurements, but the disinfectant was determined to have no cytotoxicity against these cells because a cytopathic effect (CPE) was observed under a microscope. Additionally, it was determined that no cytotoxicity occurred in the stock solution of the sample used for cytotoxicity confirmation, indicating that the detection limit was 1.3 × 10^1^ TCID_50_/mL.

**Table 2:** Evaluation of the cytotoxicity of the disinfectant.

Pathogens/cell lines	Rate of viable cells (%)^a^		Cytotoxicity (yes/no)
	Stock solution	10-Fold diluted solution	
#10: Influenza A virus/MDCK cells	114 ± 7	107 ± 4	No
#11: Feline calicivirus/CRFK cells	125 ± 4	106 ± 5	No
#12: Human adenovirus 5/Vero cells	113 ± 6	120 ± 5	No
#13: Human enterovirus 71/A549 cells	113 ± 6	120 ± 5	No

*MDCK* Madin–Darby canine kidney, *CRFK* Crandell–Rees feline kidney.

^a^Average value and standard deviation of 4 wells.

Finally, the results of experiment 3 are presented in Table 3. The skin reaction after both 30 min and 24 h in the human open patch test was completely negative for all healthy volunteers, indicating that the disinfectant is safe for human skin.

**Table 3:** Skin reaction in the human patch test.

Skin reaction	Disinfectant	
	30 min after application	24 h after removal
−	26/26	26/26
±	0/26	0/26
+	0/26	0/26
++	0/26	0/26
+++	0/26	0/26

## Discussion

Disinfection is an important method to prevent infection and cuts off infection routes. The selection of disinfectants according to the situation and environment often requires expertise and experience. Rutala and Weber^14^ listed 14 properties of an ideal disinfectant and 5 key considerations for selecting the optimal disinfectant. To date, no product that meets all of the properties for healthcare disinfection has been introduced; however, products with these properties and meeting these key considerations as much as possible are recommended as disinfectants. Recently, HAIs as well as viral pandemics, such as influenza virus and coronavirus, have caused social disturbances worldwide^4–9^. The EPA also recommends the use of disinfectants that cover the vast majority of HAIs (79.1%)^15^. We examined the bactericidal and virucidal efficacy of a newly developed disinfectant against microorganisms that can cause HAIs and pandemics and confirmed its effectiveness.

Currently, alcohol is most often used as an antiseptic, but it is not EPA-registered and is classified as a low-level disinfectant. Alcohol has a relatively broad spectrum for microorganisms but is slow to act against nonenveloped viruses. Additionally, it is difficult for alcohol to complete the required contact time with microorganisms because of its rapid evaporation, and it is not recommended for use on large surfaces because of its flammability. Rutala and Weber^2^ summarized the advantages and disadvantages of other low-level disinfectants. Based on their properties, the disinfectants not only require proper contact time with microorganisms but also must have a short kill time to achieve complete disinfection. Generally, the contact time should be longer than or equal to the kill time. Most alcohol-based solutions dry quickly, while aqueous-based disinfectants such as hypochlorite and phenolics will maintain a wet-contact time of approximately 1–2 min^14^. Our disinfectant is a nonalcoholic product made by mixing three types of metal ions (iron, zinc, and nickel) with an amino acid (L-cysteine), a surfactant (sodium lauryl sulfate), vitamin C (ascorbic acid) and an organic compound (potassium sorbate). This disinfectant rapidly inactivated almost all of the microorganisms tested. Additionally, this is a nonalcoholic liquid and contains a surfactant; hence, this may prolong the contact time with microorganisms. The metal ions are expected to be involved in sterilization. Several studies have reported that iron chloride exhibits antiviral activity in a concentration-dependent manner against herpes simplex virus type 1 (HSV-1) and bovine viral diarrhea virus and decreases the number of RNA or DNA replicates^16^. Zinc was shown to play a complementary role in enhancing bactericidal activity against the common mastitis causative pathogens *Streptococcus uberis*, *S. aureus*, and *E. coli.* Additionally, zinc has been reported to enhance virucidal activity against HSV-1 up to fourfold and mediate antiviral effects through inhibition of viral penetration or egress or progression of the intracellular phase of the viral life-cycle of transmissible gastroenteritis virus. Consequently, zinc has been shown to mediate antibacterial and antiviral effects against certain bacteria and viruses^17–19^. Some antimicrobial effects of other additives have also been reported. L-cysteine is an important amino acid and has been reported to inhibit the growth of various strains of *E. coli*^20^. Additionally, cysteine thiol groups often interact with metal ions and are involved in binding, transport, and storage of these ions in the cell^21^. Sodium dodecyl sulfate (SDS) is widely known as a representative anionic surfactant and is one of the main components of hand soaps. SDS was shown to have anti-influenza virus effects on a human influenza virus strain (H3N2). It is believed that the mechanism of inactivation of the influenza virus is an electrical interaction between the surfactant and HA proteins^22^. A former disinfectant of ours, reported by Takakuwa et al*.*^13^, includes a small amount of surfactant and has very strong anti-influenza viral activity (including avian, swine, and human). Ascorbic acid, also called vitamin C (VC), has been widely known for its antioxidant properties, immunomodulatory and anti-infectious effects since the 1930s and can scavenge damaging reactive oxygen species. Numerous reports have indicated that VC has antibacterial effects against distinct bacteria in vitro. VC concentrations of 0.31 mg/mL, 0.15 mg/mL, and 0.5 mg/mL effectively inhibited *Pseudomonas aeruginosa* (*P. aeruginosa*), *S. aureus/Enterococcus faecalis*, and *Campylobacter jejuni* growth in vitro, respectively. Furthermore, 8–16 μg/mL (i.e., low-level) VC effectively counteracted biofilm formation by MRSA. VC alone could even more effectively inhibit Salmonella growth. Several studies have reported that VC inhibited the replication of HSV-1, poliovirus type 1, and influenza virus type A, and it exhibited low-level fungistatic activity against *Candida albicans* (*C. albicans*). Thus, VC possesses antimicrobial activities that reduce the pathogenicity of bacteria, viruses, and fungi^23^. Therefore, we hypothesize that this disinfectant exerts an antimicrobial effect via the coordinated action of these ingredients. However, in this study, our disinfectant had different virucidal efficacies between norovirus (feline calicivirus) and enterovirus, which belong to a family of nonenveloped RNA viruses. It is well known that alcohol has a minimal virucidal effect and that sodium hypochlorite is effective for disinfecting these nonenveloped viruses. Sato et al. reported that low-pH alcohol (acid-alcohol) had virucidal efficacy against human norovirus, indicating enhancement of the virucidal effect of alcohol by acidification^24^. On the other hand, enteroviruses are well recognized to be stable under acidic conditions. The virucidal efficacy against enterovirus 71 has been reported to be higher under alkaline conditions (pH 8.2)^25^. The differences in virucidal efficacy among nonenveloped viruses may be pH dependent.

The proper selection of a disinfectant requires taking into account not only antimicrobial activity and contact time but also a lack of harmful effects on the human body. Safety involves several components, including the toxicity, flammability and compatibility of the substance and the use of personal protective equipment. The disinfectant should be nontoxic and harmless to users. The least toxic product should be selected. It is generally known that nickel, which is contained in our disinfectant, is a common allergen responsible for allergic contact dermatitis^26^. However, the human skin patch test confirmed safety in all subjects. Additionally, this disinfectant showed little acute cytotoxicity in vitro. In the future, we plan to evaluate effects on the mucous membrane and eyes as a further safety assessment.

Our new disinfectant is a next-generation product, and its in vitro antimicrobial effects and safety were confirmed in this study. However, its mechanism of action, antimicrobial effects under other conditions (in the presence of organic substances, etc.), and clinical efficacy remain unclear. Therefore, it is necessary to further study the antimicrobial effects and safety of this disinfectant, but this is a product with many possibilities.

## Methods

### Disinfectant

To prepare the disinfectant, the bacteriostatic action was verified for each component, and the optimum concentration was confirmed. These components were mixed to adjust the concentration having the strongest bactericidal action. Additionally, it was confirmed that the bactericidal effect was further enhanced by adjusting the pH to approximately 3. Therefore, the new disinfectant (FEION) was prepared as follows. First, solution A was made by dissolving 0.96 g of FeCl_3_6H_2_O, 0.25 g of ZnSO_4_ and 0.18 g of NiSO_4_7H_2_O in 200 mL of distilled water (Otsuka Pharmaceutical). Second, solution B was prepared by dissolving 1 g of l-cysteine, 0.1 g of ascorbic acid, 0.05 g of potassium sorbate, and 0.1 g of sodium lauryl sulfate in 800 mL of distilled water. Finally, solutions A and B were mixed, and 3 N HCl was added to this mixture to adjust it to pH 2.3–2.5. This new disinfectant was a colorless and transparent liquid and had less metallic odor than the previous ones. Additionally, a patent application for this disinfectant has been submitted to the Japan Patent Office (patent number: JP 5327218).

### Experiment 1

#### Preparation of bacterial test solutions

The efficacy of this disinfectant was tested on 9 bacteria (#1: *E. coli*, #2: *E. coli* (O157), #3: *P. aeruginosa*, #4: *Salmonella enterica* (*S. enterica*), #5: *S. aureus*, #6: *S. aureus* (MRSA), #7: *Vibrio parahaemolyticus* (*V. parahaemolyticus*), #8: *C. jejuni*, and #9: *C. albicans*) and 4 viruses (#10: influenza A virus, H1N1, #11: feline calicivirus (a norovirus surrogate), #12: human adenovirus 5, and #13: human enterovirus 71) at the Kitasato Research Center for Environmental Science, a third-party institution. The bacteria tested are presented in Table 1. First, the cryopreserved bacterial strains #1–#9 were inoculated and cultured under the conditions shown in Supplementary Table S2. Then, the grown colonies were scraped, suspended in sterile ion-exchanged water (#1–#6, #9), 3% NaCl (#7) or KH_2_PO_4_ (3.4% solution mixed with water at 1:800, Wako) (#8) to prepare a mixture at approximately 10^7^ colony-forming units per milliliter (CFU/mL), which was used as the bacterial test solution. The initial inoculum size of *E. coli*, *E. coli* O157, *P. aeruginosa*, *S. enterica*, *S. aureus*, MRSA, *V. parahaemolyticus*, *C. jejuni*, and *C. albicans was* 5.2 × 10^7^ CFU/mL, 4.6 × 10^7^ CFU/mL, 6.5 × 10^7^ CFU/mL, 7.0 × 10^7^ CFU/mL, 5.7 × 10^7^ CFU/mL, 4.8 × 10^7^ CFU/mL, 5.0 × 10^7^ CFU/mL, 2.5 × 10^7^ CFU/mL, and 7.0 × 10^7^ CFU/mL, respectively.

#### Preparation of viral test solutions

The viruses tested are presented in Table 1. First, #10 was inoculated into embryonated specific-pathogen-free hen eggs and cultured at 35.5 °C for 2 days. Then, the allantoic fluid was collected and concentrated with an ultrafiltration membrane, which was followed by sucrose density gradient centrifugation (centrifugation condition: 108,000×*g*, 4 °C, for 3 h) to obtain a viral solution. Viruses #11-#13 were infected into Crandell-Rees feline kidney (CRFK), A549 and Vero cells, respectively, and when approximately 90% or more of the cell-cultured area showed a CPE, the cells were cryopreserved in a freezer at − 30 °C. Thereafter, a freeze–thaw operation was performed, and the supernatant obtained by centrifugation at 2380×*g* for 10 min (min) was collected and concentrated by the ultrafiltration membrane. Viruses #12 and #13 were preserved as virus solutions at this stage. The collected solution (#11) was further concentrated by a sucrose cushion method (centrifugation conditions: 108,000×*g*, 4 °C, for 3 h) and finally preserved as a virus solution. For the test, all of the virus solutions (#10–13) were diluted tenfold with Dulbecco’s phosphate-buffered saline (–) (PBS, Nissui Pharmaceutical) and used.

#### Evaluation of bactericidal efficacy (suspension test)

The suspension test was performed according to the 17th revised Japanese Pharmacopoeia 4.05 (JP17, English version, Reference Information, Microbiological Examination of Non-sterile Products, 121–130)^27^. A test bacterial solution (0.1 mL) was added to disinfectant (10 mL), and mixed with a tube mixer, and incubated at 25 ± 2 °C for 0 (initial), 5 min, and 30 min. After the action for a predetermined time, the mixture (1 mL) was added to inactivator (9 mL) (SCDLP bouillon medium (SCDLP), Eiken Chemical Co., Ltd., or SCDLP containing 0.25% NaCl, data in the Supplementary Table S1) to stop the bactericidal action, and this solution was prepared as a sample solution for measuring the number of bacteria. Distilled 0.85% NaCl (Wako) was used in place of the disinfectant for the initial time and the control, and 3% NaCl was used for *Vibrio parahaemolyticus*.

#### Evaluation of virucidal efficacy (virus inactivation test)

The virus inactivation test was performed according to ASTM international E1052 method (Standard Test Method to Assess the Activity of Microbicides against Viruses in Suspension). After dispensing 0.9 mL of the disinfectant into a test tube, 0.1 mL of a virus solution was added and mixed. Each mixture was allowed to act at room temperature for a predetermined time. To stop the action of the disinfectant, we added 0.1 mL of the mixture to 9.9 mL of the inactivator (SCDLP) to dilute the sample. This solution was used as a stock solution for measuring viral infection titers. In addition, PBS was used in place of the disinfectant for the action at time 0 (initial) and in the control.

#### Inactivator validation

Since our disinfectant is at a low pH, it was judged that the action cannot be stopped in MEM medium, which has almost no pH-buffering capacity. Therefore, the effectiveness of the inactivator SCDLP used to stop the bactericidal and virucidal activities of the disinfectant was verified. The methods and results are presented in Supplementary Table S1.

#### Measurements of bacterial counts and viral infection titers

The medium and reagents used are shown in Supplementary Table S3. A set of tenfold serial dilutions was prepared for the sample solution with each medium and reagent. Then, each of the sample stock solutions and the diluent (1 mL) was transferred to a Petri dish; after mixing with each medium, these were solidified and cultured under the conditions shown in Supplementary Table S3. The sample stock solution of Campylobacter (#8) was also diluted, smeared and cultured as shown in Supplementary Table S3. Finally, the number of colonies that grew was counted, and the number of test bacteria per 1 mL of the test product was determined. Bacterial count measurements were performed using the plate-count method. The choice of method was determined based on the bacterial species. Bacteria #1–#7/#9 and #8 were counted using the pour-plate method and surface-spread method, respectively. The lower limits of quantification were 10 CFU/mL and 100 CFU/mL, respectively.

The viral infection titers were measured using the 50% tissue culture infectious dose (TCID_50_) method. Cells for measuring the viral infection titers were seeded in a 96-well plate and cultured in a CO_2_ incubator (MCO-20AIC, SANYO) for 4 days. Next, a stock solution of the viral infection titer sample was serially diluted tenfold with PBS. Each well without the cultured solution was inoculated with 25 μL of a stock solution for measuring infectious titers or a sample diluted tenfold in PBS, and the cells were infected with the virus at 37 °C for an hour. Thereafter, the inoculated virus solution was removed, 0.1 mL of medium for virus culture (see Supplementary Table S2 for details) were added per well, and the cells were cultured in a CO_2_ incubator at 37 °C (culture periods, see Supplementary Table S3 for details). After culturing, the CPE generated by virus multiplication was observed under a microscope, and the viral infection titers were determined using the Reed-Muench method and expressed as TCID_50_/mL.

#### Calculation of microbial log reduction values

A log reduction value (LRV) was used as an evaluation parameter for judging the bactericidal and virucidal activities according to the JP17 and European Norm (EN) 14,476: 2013 + A1: 2015^28^. The formula for calculating LRVs is log_10_ (initial bacterial count of a control divided by the bacterial count after the action of the disinfectant or initial viral infection titer of a control divided by the viral infection titer after the action of the disinfectant). Note that LRVs are represented to one decimal place (rounded down). Here, an LRV of 3 or greater calculated using the numbers of bacteria before and after the action of the disinfectant (usually for 5–15 min) was used to define the disinfectant as "effective" against bacteria. Then, LRVs of 4 or greater calculated using the viral titers before and after the action of the disinfectant (usually for 15 s to 5 min) indicated that the disinfectant was "effective" for viruses.

### Experiment 2

The cytotoxicity of this disinfectant for each cultured cell line (for the cell lines used for determining infectious titer, see Table 1 for details) was investigated. The cytotoxicity test was generally conducted according to ASTM E1052. After adding PBS (0.1 mL) to the disinfectant (0.9 mL), a solution diluted 100-fold with SCDLP was used for measurements of cytotoxicity. This stock solution and a solution diluted tenfold with PBS were inoculated with 25 μL per well of cell lines previously cultured in a monolayer in a 96-well plate and then incubated at 37 °C for an hour in a CO_2_ incubator. Then, the inoculum was removed, and an infectious titer measurement medium (0.1 mL per well) was added, followed by culturing in a CO_2_ incubator. Thereafter, the cells were stained with crystal violet, and cytotoxicity was evaluated by the degree of staining of each well. The cytotoxicity was determined by calculating the viable cell rate (%). The viable cell rate, when cells were cultured in PBS, was defined as 100%, and values less than 50% indicated cytotoxicity when samples were cultured in the above disinfectant solution. Additionally, when the CPE of the virus could be determined by microscopic observation even when the viable cell rate was less than 50%, the disinfectant was determined to have no cytotoxicity.

### Experiment 3

**Figure 1:**
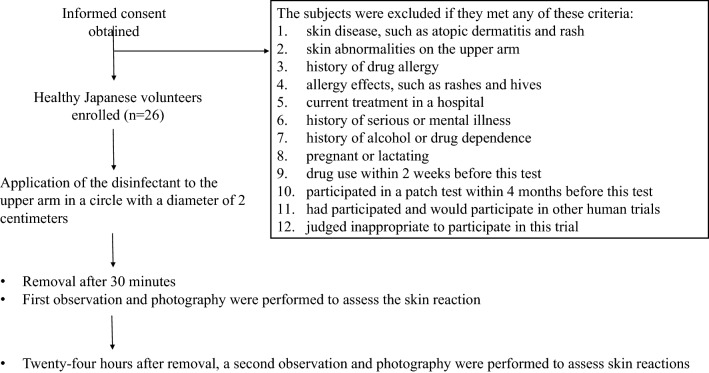
Flow chart of patient selection and patch test schedule.

This safety study was performed by the human open patch test at the DRC corporation. A total of twenty-six healthy Japanese (5 men and 21 women) were enrolled in this study. The median age of the study population was 42 years (range 27–59). This study conformed to the Helsinki Declaration and local legislation and was approved by the Research Ethics Committee of the DRC corporation (Examination number: BCC190913-3, Approval date 9/13/2019). We confirmed that informed consent was obtained from all participants. The schedule and exclusion criteria are shown in Fig. 1. Grading of the skin reaction in the patch test was evaluated at 30 min and 24 h after application according to the Japanese standards of the Japanese Dermatological Association Contact Dermatitis Clinical Practice Guidelines (2009): no reaction (−); slight erythema (+); erythema (+); erythema with edema (++); and erythema with vesicles and/or papules (+++)^29^. Additionally, the DRC corporation stores more than 1000 photographs every year to assist in the judgment of skin reactions.

## Supplementary Information

Supplementary Information

## Data Availability

Please contact the author for data requests.
