# Supplementary material for: The effectiveness of a nonalcoholic disinfectant containing metal ions, with broad antimicrobial activity
Source: Sci Rep. 2021 Jan 13;11:1072. doi: 10.1038/s41598-020-80443-6 (PMC7806819; doi:10.1038/s41598-020-80443-6)
Supplement: Supplementary file 1 — Supplementary Information [file 41598_2020_80443_MOESM1_ESM.docx]

**The effectiveness of a nonalcoholic disinfectant containing metal ions, with broad antimicrobial activity**

Tokuhiro Matsubara^a^, Shuichi Maki^b,c^, Yukiko Toshimori^b,c^

^a^Department of Gastroenterology, Tsukaguchi Hospital, 6-8-1 Minamitsukaguchi-cho, Amagasaki, Hyogo 661-0012 Japan

^b^Yui-Nozomi Hospital, 95 Fushimido, Tondabayashi, Osaka 584-0055 Japan

^c^Shiniryouzaidan, 3-7-4 Matsuzaki-cho, Abeno-ku, Osaka 545-0053 Japan

**SUPPLEMENTARY MATERIAL**

**Supplementary Table S1.** Test to confirm inactivator efficacy.

| Pathogens | | Inactivator^a)^  (100-fold dilution) | Number of bacteria (CFU/ml) | | Ratio of B to A^b)^ | Judgment^c)^ |
| --- | --- | --- | --- | --- | --- | --- |
|  |  |  | A | B |  |  |
| #1 | *E. coli* | SCDLP | 8.5×10 | 8.6×10 | 101 | effective |
| #2 | *E. coli (O157)* | SCDLP | 6.6×10 | 7.3×10 | 111 | effective |
| #3 | *P. aeruginosa* | SCDLP | 8.7×10 | 9.3×10 | 107 | effective |
| #4 | *Salmonella enterica* | SCDLP | 1.3×10^2^ | 1.2×10^2^ | 92 | effective |
| #5 | *S. aureus* | SCDLP | 8.9×10 | 9.1×10 | 102 | effective |
| #6 | *MRSA* | SCDLP | 9.5×10 | 1.0×10^2^ | 105 | effective |
| #7 | *Vibrio parahaemolyticus* | SCDLP+2.5% NaCl | 7.2×10 | 8.6×10 | 119 | effective |
| #8 | *Campylobacter jejuni* | SCDLP | 2.2×10^2^ | 2.8×10^2^ | 127 | effective |
| #9 | *Candida albicans* | SCDLP | 1.4×10^2^ | 1.4×10^2^ | 100 | effective |

| Pathogens | | Inactivator^a)^  (100-fold dilution) | Viral infection titer (TCID_50_/ml) | | Log_10_ (A/B) | Judgment^d)^ |
| --- | --- | --- | --- | --- | --- | --- |
|  |  |  | A | B |  |  |
| #10 | Influenza A virus | SCDLP | 8.9×10^3^ | 7.0×10^3^ | 0.1 | effective |
| #11 | Feline calicivirus | SCDLP | 1.0×10^4^ | 1.1×10^4^ | 0 | effective |
| #12 | Human adenovirus 5 | SCDLP | 1.5×10^3^ | 1.3×10^3^ | -0.2 | effective |
| #13 | Human enterovirus 71 | SCDLP | 1.1×10^3^ | 2.1×10^3^ | -0.2 | effective |

#1: *Escherichia coli* NBRC3972, #2: *Escherichia coli* (157: H7), #3: *Pseudomonas aeruginosa* NBRC13275, #4: *Salmonella enterica* subsp. *enterica* NBRC3313, #5: *Staphylococcus aureus* NBRC12732, #6: *Staphylococcus aureus* (MRSA) IID1677, #7: *Vibrio parahaemolyticus* NBRC12711, #8: *Campylobacter jejuni* subsp. *jejuni* JCM2013, #9: *Candida albicans* NBRC1594, #10: Influenza A virus, H1N1, A/PR/8/34, ATCC VR-1469, #11: Feline calicivirus, F-9, ATCC VR-782 (a norovirus surrogate), #12: Human adenovirus 5, Adenoid 75, ATCC VR-5, #13: Human enterovirus 71, H, ATCC VR-1432, SCDLP: SCDLP bouillon medium (Eiken Chemical Co., Ltd.), A: control (sterilized water), B: disinfectant, ^a)^: reaction at room temperature for 20 min, ^b)^: B/A×100, ^c)^: According to the 17th revised Japanese Pharmacopoeia 4.05-I-3.5, the criterion range was set as 50-200% (“effective”), ^d)^: The test sample was determined to be effective when the infectivity titer did not decrease by 0.5 log_10_ or more compared to that of the control.

**Supplementary Table S2.** Preparation of the bacterial test solutions.

| Pathogens | Medium | Culture conditions | Medium replacement | Reculture conditions |
| --- | --- | --- | --- | --- |
| #1-#6 | TSA | 36+2°C, 24 hours | same medium | 36+2°C, 18 hours |
| #7 | TSA containing 2.5% NaCl | 36+2°C, 24 hours | same medium | 36+2°C, 18 hours |
| #8 | BA containing 5% heat-inactivated horse serum | 36+2°C, 3 days* | BB containing 5% heat-inactivated horse serum | 36+2°C, 2 days* |
| #9 | PDA | 26+2°C, 2 days | none | none |

TSA: tryptic soy agar (Difco), BA: Brucella agar (BBL), 5% heat-inactivated horse serum (GIBCO), PDA: potato dextrose agar (Nissui Pharmaceutical), BB: Brucella broth (BBL), NaCl: sodium chloride (Wako), *: culture under microaerobic conditions.

**Supplementary Table S3.** Measuring methods for the bacterial counts and viral infection titers.

| Pathogens | Diluted solutions | Mixture | Volume added | Culture conditions |
| --- | --- | --- | --- | --- |
| #1-#6 | 0.85% NaCl | TSA | 20 mL | 36±2°C, 43 hours |
| #7 | 3% NaCl | TSA containing 2.5% NaCl | 20 mL | 36±2°C, 48 hours |
| #8 | KH_2_PO_4_ | Smear on BA containing 5% heat-inactivated horse serum |  | 36±2°C, 3 days* |
| #9 | 0.85% NaCl | PDA | 20 mL | 26±2°C, 4 days |

| Pathogens | Medium | Culture conditions |
| --- | --- | --- |
| #10 | MEM containing 0.42% BSA and 5 µg/ml trypsin | 4 days |
| #11 | DMEM containing 1% FBS | 4 days |
| #12 | DMEM containing 0.2% FBS | 6 days |
| #13 | DMEM containing 1% FBS | 6 days |

NaCl: sodium chloride (Wako), TSA: tryptic soy agar (Difco), BA: Brucella agar (BBL), 5% heat-inactivated horse serum (GIBCO), PDA: potato dextrose agar (Nissui Pharmaceutical), BB: Brucella broth (BBL), *: culture under microaerobic conditions, MEM: modified Eagle’s medium (Nissui Pharmaceutical), BSA: bovine serum albumin (Sigma Aldrich), trypsin (Sigma Aldrich), DMEM: Dulbecco’s modified Eagle’s medium (Nissui Pharmaceutical), FBS: fetal bovine serum (Sigma Aldrich).
